# Thermal Management of Serpentine Flexible Heater Based on the Orthotropic Heat Conduction Model

**DOI:** 10.3390/mi13040622

**Published:** 2022-04-15

**Authors:** Zhao Zhao, Jin Nan, Min Li

**Affiliations:** 1 Institute of Solid Mechanics, Beihang University (BUAA), Beijing 100191, China; ouczhaozhao@163.com; 2 School of Mechanics and Civil Engineering, China University of Mining and Technology, Beijing 100083, China; nanjincn@163.com; 3 Aircraft and Propulsion Laboratory, Ningbo Institute of Technology, Beihang University (BUAA), Ningbo 315100, China

**Keywords:** orthotropic heat conduction, flexible heater, serpentine heat source, thermal uniformity

## Abstract

Flexible heaters can perfectly fit with undevelopable surfaces for heating in many practical applications such as thermotherapy, defogging/deicing systems and warming garments. Considering the requirement for stretchability in a flexible heater, certain spacing needs to be retained between serpentine heat sources for deformation which will inevitably bring critical challenges to the thermal uniformity. In order to reconcile these two conflicting aspects, a novel method is proposed by embedding the serpentine heat source in orthotropic layers to achieve comprehensive performance in stretchability and uniform heating. Such a scheme takes advantage of the ability of orthotropic material to control the heat flow distribution via orthotropic thermal conductivity. In this paper, an analytical heat conduction model with orthotropic substrate and encapsulation is calculated using Fourier cosine transform, which is validated by finite element analysis (FEA). Meanwhile, the effects of the orthotropic substrate or encapsulation with different ratios of thermal conductivity and the geometric spacing on the thermal properties are investigated, which can help guide the design and fabrication of flexible heaters to achieve the goal of uniform heating.

## 1. Introduction

A flexible heater, as a novel heating device, is widely used in heating undevelopable curved surfaces, such as in cutaneous wound healing [1], subcutaneous tumor treatment [2], thermal releasing drug [3], defogging/deicing systems [4,5], heatable curved windows [6] and so on.

Recently, there has been a variety of advancements in developing flexible heaters based on stretchable polymers with conductive compositions [7]. For example, a metal nano network [8], copper-plated fibers [9] and graphite [10] as flexible heaters have been investigated. However, the slippage of fiber in these heaters will result in higher electrical resistance in the local position under deformation, which causes extraordinarily higher temperatures at this point (i.e., called “hot spots”) [9]. To avoid this problem, periodical metal wire as a heat source was developed in flexible heaters. Jang [11] investigated a stretchable and uniform Kirigami heater for heating the wrist and elbow with large motions, where aluminum-doped conductive paper is processed into a stretchable shape. Chen et al. [12] adopted a copper mesh heat source as a skin patch for thermotherapy, which was shown to be highly conformable to the skin at high stretching.

On the other hand, a stretchable design for heat sources requires certain spacing to keep deformability, which will result in uneven temperatures at these positions. In order to keep balance with better mechanical compatibility and temperature uniformity, a heat source is designed with a serpentine structure [13,14,15] embedded in orthotropic substrate and encapsulation. Significantly, the orthotropic model based on a multilayered composite material (i.e., thermal metamaterials) can control the temperature and the manipulation of thermal flux via thermal anisotropy [16,17,18]; it can help achieve the goal of temperature uniformity. As for thermal management in flexible electronics, many analytical heat conduction models have been investigated in previous studies. For example, a heat conduction model of a single and an infinite number of rectangular heat sources in an isotropic model was developed [19,20] based on the transfer matrix method and Fourier heat transfer. Additionally, the Hankel transform was used to solve the heat conduction of a point heat source in flexible electronic devices [21].

Compared to the regular heat sources in previous studies, the analytical orthotropic heat conduction model with the complex serpentine heat source is developed in this paper. The thermal field of the model is calculated using Fourier cosine transform and linear superposition of a rectangular heat source in Section 2, which is validated by Finite element analysis (FEA) in Section 3. Meanwhile, the effects of different ratios of thermal conductivity in substrate/encapsulation and different spacing of the serpentine heat source on the thermal properties are discussed. Section 4 presents the conclusion.

## 2. Analytical Modeling

**Figure 1:**
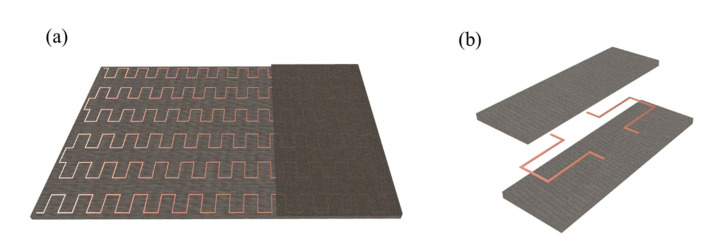
(**a**) Schematic diagram of serpentine heat source embedded in orthotropic membranes; (**b**) a single period of the periodic structure.

Figure 1a shows the flexible heater with periodic serpentine heat sources embedded in the orthotropic substrate and encapsulation. Due to periodicity, only a unit cell is investigated, as shown in Figure 1b.

**Figure 2:**
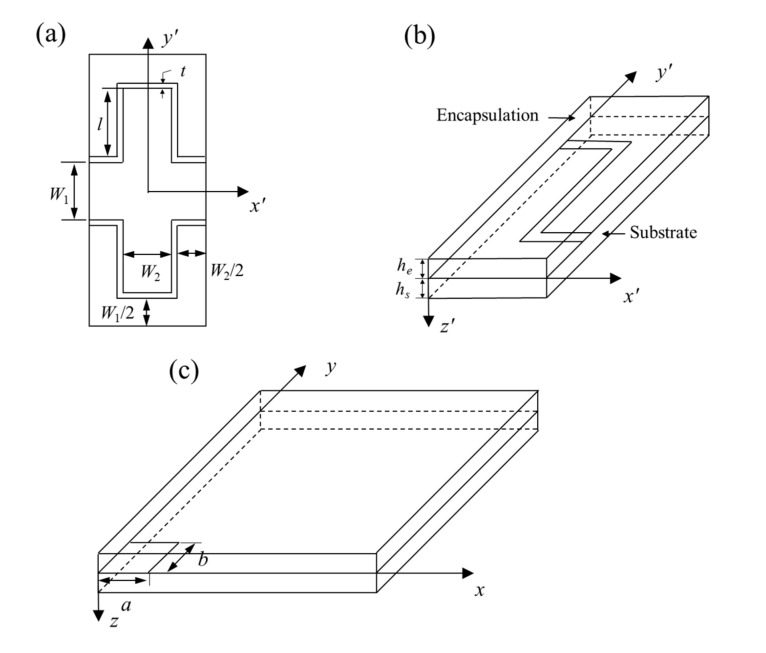
Schematic diagrams of (**a**) a single period and (**b**) a quarter of the model; (**c**) a rectangle heat source embedded in a quarter of the model with infinite size in the plane.

As for thermal properties in the orthotropic model, the thermal conductivities in layered thermal metamaterials consist of two kinds of material yield, [16,22]

(1) $$k^{m}=\frac{k_{A}+k_{B}}{2},k^{n}=\frac{2k_{A}k_{B}}{k_{A}+k_{B}}$$

where *k^m^* and *k^n^* are the thermal conductivity along in-plane and off-plane (i.e., the laying direction of different materials) directions, respectively. *K_A_* and *k_B_* represent the thermal conductivity of layered materials *A* and *B*. The geometric parameters of a period of serpentine heat source, including the vertical space *W*_1_, the horizontal space *W*_2_, the width *t* and the length of straight part *l*, are shown in Figure 2a. Due to symmetry, only a quarter of the heat conduction model consisting of encapsulation, substrate and the heat source is studied in Figure 2b. Compared with the whole thickness of the model, the ultrathin serpentine heat source (thickness of copper selected as 2.6 μm [23]) based on micro-nano machining is negligible in this paper, which can be regarded as a planar heat source in heat conduction [24].

In this paper, the temperature increase in the infinite plane model with any arbitrary size of a rectangular planar heat source is calculated at first (shown in Figure 2c), then the linear superposition principle is used to obtain the temperature increase distribution of the serpentine structure in the unit cell. Figure 2c presents a quarter of the model with infinite size in the plane, where the coordinate system is established at the center of the rectangle planar heat source as the origin, the *x*-axis and *y*-axis follow the *a* and *b* sides, respectively, and the *z*-axis points from the heat source to the substrate. The temperature in the system is denoted by *T*_i_(*x,y,z*), and thus, the temperature increase in the model is denoted by *θ_i_* = *T_i_* − *T_∞_* from the ambient temperature *T_∞_*. The heat conduction equation in the orthotropic model gives

(2) $$k_{i}^{x}\frac{\partial^{2}\theta_{i}}{\partial x^{2}}+k_{i}^{y}\frac{\partial^{2}\theta_{i}}{\partial y^{2}}+k_{i}^{z}\frac{\partial^{2}\theta_{i}}{\partial z^{2}}=0$$

where *i* can be *e* (encapsulation) and *s* (substrate). *k^x^*, *k^y^* and *k^z^* denote the thermal conductivity in the *x*, *y* and *z*-direction, respectively. At the top and bottom surfaces of the structure, the natural convection condition yields

(3) $${\left.-k_{s}^{z}\frac{\partial \theta_{s}}{\partial z}\right|}_{z=h_{s}}=h_{0}\theta_{s}$$

(4) $${\left.k_{e}^{z}\frac{\partial \theta_{e}}{\partial z}\right|}_{z=-h_{e}}=h_{0}\theta_{e}$$

where *h*_0_ is the coefficient of heat convection. Considering the continuity of temperature increase and heat flux on the interface with the heat source between the substrate and encapsulation, it can be expressed by

(5) $${\left.\theta_{e}\right|}_{z=0^{-}}={\left.\theta_{s}\right|}_{z=0^{+}}$$

(6) $${\left.k_{e}^{z}\frac{\partial \theta_{e}}{\partial z}\right|}_{0^{-}}-{\left.k_{s}^{z}\frac{\partial \theta_{s}}{\partial z}\right|}_{0^{+}}=\left\{\begin{array}{l} Q_{0},\;(x,y)\in D\\ 0,(x,y)\notin D\;\; \end{array}\right.$$

where *D* and *Q*_0_ are the region and the heat flux density of the rectangle planar heat source. Using the Fourier cosine transform, the temperature increase in the model can be written as

(7) $$\bar{\theta_{i}}(\alpha ,\beta ,z)=\int_{0}^{\infty }\int_{0}^{\infty }\theta_{i}(x,y,z)\cos(\alpha x)\cos(\beta y)dxdy$$

which is used to solve the definite solutions of steady heat conduction in the model. Therefore, Equations (2)–(6) based on the Fourier cosine transform become

(8) $$\frac{d^{2}\bar{\theta_{i}}}{dz^{2}}-\left(\frac{\alpha^{2}k_{i}^{x}+\beta^{2}k_{i}^{y}}{k_{i}^{z}}\right)\bar{\theta_{i}}=0$$

(9) $${\left.-k_{s}^{z}\frac{d\bar{\theta_{s}}}{dz}\right|}_{z=h_{s}}=h_{0}\bar{\theta_{s}}$$

(10) $${\left.k_{e}^{z}\frac{d\bar{\theta_{e}}}{dz}\right|}_{z=-h_{e}}=h_{0}\bar{\theta_{e}}$$

(11) $${\left.\bar{\theta_{e}}\right|}_{z=0^{-}}={\left.\bar{\theta_{s}}\right|}_{z=0^{+}}$$

(12) $${\left.k_{e}^{z}\frac{d\theta_{e}}{dz}\right|}_{0^{-}}-{\left.k_{s}^{z}\frac{d\theta_{s}}{dz}\right|}_{0^{+}}=\frac{Q_{0}\sin(\alpha a)\sin(\beta b)}{\alpha \beta }$$

The general solution in Equation (8) can be expressed by

(13) $$\bar{\theta_{i}}=A_{i}e^{\sqrt{\frac{\alpha^{2}k_{i}^{x}+\beta^{2}k_{i}^{y}}{k_{i}^{z}}}z}+B_{i}e^{-\sqrt{\frac{\alpha^{2}k_{i}^{x}+\beta^{2}k_{i}^{y}}{k_{i}^{z}}}z}$$

The coefficients *A_s_*, *A_e_*, *B_s_* and *B_e_* in Equation (13) are determined by Equations (9)–(12), which can be obtained by

(14) $$\begin{array}{l} A_{s}=\frac{1}{\alpha \beta }\frac{\sin(a\alpha )\sin(b\beta )\left(h_{0}-C_{1}\right)\left(\left(h_{0}-C_{2}\right)e^{-2h_{e}\xi_{e}}-C_{2}-h_{0}\right)Q_{0}}{\left[\begin{array}{l} \left(\left(C_{1}-C_{2}\right)\left(h_{0}+C_{1}\right)e^{2h_{s}\xi_{s}}+\left(C_{1}+C_{2}\right)\left(h_{0}-C_{1}\right)\right)\left(C_{2}-h_{0}\right)e^{-2h_{e}\xi_{e}}\\ +\left(\left(C_{1}+C_{2}\right)\left(h_{0}+C_{1}\right)e^{2h_{s}\xi_{s}}+\left(C_{1}-C_{2}\right)\left(h_{0}-C_{1}\right)\right)\left(h_{0}+C_{2}\right) \end{array}\right]},\\ A_{e}=\frac{1}{\alpha \beta }\frac{\sin(a\alpha )\sin(b\beta )\left(h_{0}+C_{2}\right)\left(\left(h_{0}+C_{1}\right)e^{2h_{s}\xi_{s}}+C_{1}-h_{0}\right)Q_{0}}{\left[\begin{array}{l} \left(\left(C_{1}+C_{2}\right)\left(h_{0}+C_{2}\right)-\left(C_{1}-C_{2}\right)\left(h_{0}-C_{2}\right)e^{-2h_{e}\xi_{e}}\right)\left(h_{0}+C_{1}\right)e^{2h_{s}\xi_{s}}\\ -\left(\left(C_{1}+C_{2}\right)\left(h_{0}-C_{2}\right)e^{-2h_{e}\xi_{e}}-\left(C_{1}-C_{2}\right)\left(h_{0}+C_{2}\right)\right)\left(h_{0}-C_{1}\right) \end{array}\right]},\\ B_{s}=-\frac{A_{s}\left(h_{0}+C_{1}\right)e^{2h_{s}\xi_{s}}}{h_{0}-C_{1}},\\ B_{e}=-\frac{A_{e}\left(h_{0}-C_{2}\right)e^{-2h_{e}\xi_{e}}}{h_{0}+C_{2}} \end{array}$$

where

(15) $$\xi_{i}=\sqrt{\frac{\alpha^{2}k_{i}^{x}+\beta^{2}k_{i}^{y}}{k_{i}^{z}}},\;\;C_{1}=\xi_{s}k_{s}^{z},\;\;C_{2}=\xi_{e}k_{e}^{z}$$

So far, the temperature increase in the model can be calculated based on inverse Fourier cosine transform, which gives

(16) $$\theta_{i}\left(x,y,z\right)=\frac{4}{\pi^{2}}\int_{0}^{\infty }\int_{0}^{\infty }\bar{\theta_{i}}\left(\alpha ,\beta ,z\right)\cos(\alpha x)\cos(\beta y)d\alpha d\beta$$

The serpentine heat source shown in Figure 1a can be divided into many rectangular heat sources. According to the relative coordinates (*x_j_*, *y_j_*) of the geometric center point of each rectangular heat source in the *x’*-*y’*-*z’* coordinate system shown in Figure 2b, the temperature increase in a unit cell can be obtained through linear superposition of the surrounding heat source, which can be written as

(17) $$\theta_{i}\left(x,y,z\right)=\frac{4}{\pi^{2}}\sum_{j=1}^{\infty }\int_{0}^{\infty }\int_{0}^{\infty }\bar{\theta_{i}}\left(\alpha ,\beta ,z\right)\cos\left(\alpha \left(x-x_{j}\right)\right)\cos\left(\beta \left(y-y_{j}\right)\right)d\alpha d\beta$$

## 3. Results and Discussion

In this section, the orthotropic heat conduction model with the serpentine heat source is verified by FEA. Additionally, the effects of geometric spacing and the orthotropic thermal conductivity on the thermal properties are investigated. The encapsulation is selected as polyurethane (PU) with the thermal conductivity 0.19 W/m/K [25], and PDMS is used as substrate with the thermal conductivity 0.17 W/m/K [26]. The natural air convection coefficient *h*_0_ on the top and bottom surfaces is 15 W/m^2^/K. The power density is set as *Q*_0_ = 10 mW/mm^2^. As for the geometric parameters of the structure, they are selected as: the vertical space *W*_1_ = 5 mm, the horizontal space *W*_2_ = 3 mm, the width *t* = 0.2 mm, the length of straight part *l* = 5 mm and the thickness of substrate and encapsulation *h_e_* = *h_s_* = 1 mm. In FEA, 2.5 million DC3D8 heat transfer elements are dispersed in the ABAQUS software and obtain the convergence result.

**Figure 3:**
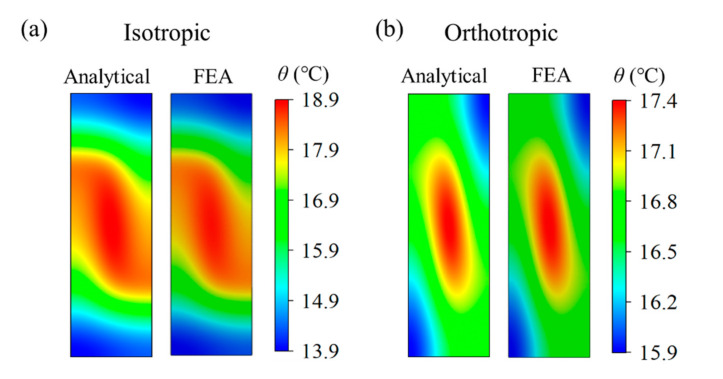
Comparison of the analytical and FEA temperature field on the bottom surface of a quarter of the unit cell based on (**a**) the isotropic and (**b**) orthotropic substrate.

As a wearable heater in the future, the temperature distribution on the bottom surface of the substrate is noteworthy because it is in contact with the target object, such as the skin. Figure 3 shows the thermal fields at the bottom surface in a quarter of the unit cell (as shown in Figure 2b), which are calculated using Equation (17) based on the linear superposition of the surrounding heat source. Figure 3a shows the comparison of the temperature field between the analytical and FEA in a quarter of the unit isotropic cell with *k_s_^x^* = *k_s_^y^* = *k_s_^z^* = 0.17 W/m/K in the substrate. It can be seen that the temperature distribution is uneven along the *y-*direction because of the larger spacing *W*_1_ between serpentine heat sources. In general, the designed vertical space *W*_1_ is larger than the horizontal space *W*_2_ if the stretchable design of structure is along the horizontal direction (Figure 1), because most of the structures will shrink along the vertical direction based on the positive Poisson’s effect under horizontal stretching. To improve the temperature uniformity, the *k_s_^y^* is set as 1.7 W/m/K and other parameters are fixed, which is based on the different materials in the substrate laying along the *y*-direction (i.e., off-plane direction) in Equation (1). Figure 3b illustrates that the orthotropic substrate with higher *k_s_^y^* has a positive influence on the temperature uniformity, which is validated by FEA.

**Figure 4:**
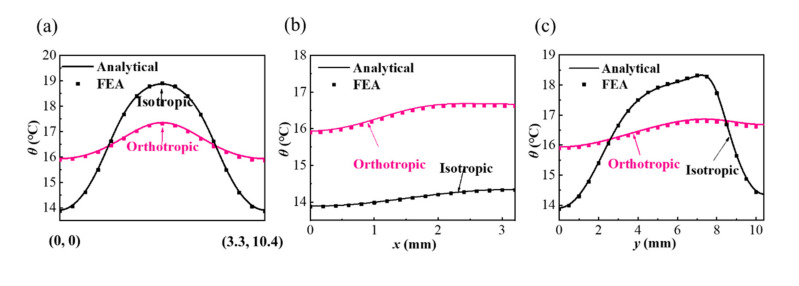
The comparisons of temperature increase along (**a**) the line from (0, 0) to (3.2, 10.4), (**b**) *y* = 2.5 and (**c**) *x* = 0 on the bottom surface a quarter of unit cell between the analytical results and FEA.

In order to show more direct comparison results, the temperature distributions along the different paths on the bottom surface of a quarter of the unit cell based on the isotropic and orthotropic substrate are shown in Figure 4. The temperature increase on the line starting from the coordinate origin (0, 0) to the coordinate (3.2, 10.4) of the upper right corner of a quarter model is calculated in Figure 4a, which includes the maximum and the minimum temperature in the model. It can be observed that the orthotropic substrate with higher *k_s_^y^* effectively reduces the temperature difference. From Figure 4b, the temperature difference between the isotropic and orthotropic substrates is close along the *x* direction (*y* = 2.5 mm); nevertheless, there is a great difference along the *y*-direction (*x* = 0), as shown in Figure 4c. This is because the higher thermal conductivity *k_s_^y^* in the *y*-direction can help obtain the uniform temperature distribution along the vertical space *W*_1_.

**Figure 5:**
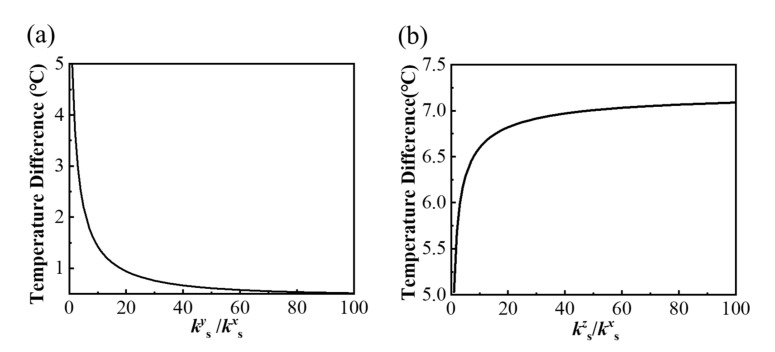
Effects of the orthotropic substrate with different ratios of (**a**) *k_s_^y^_/_k_s_^x^* and (**b**) *k_s_^z^_/_k_s_^x^* on the thermal uniformity of the bottom surface of the model.

The variable thermal conductivity in different directions of the substrate can be used for thermal management in different purposes via changing the laying direction of materials. For example, a better uniform temperature distribution can be achieved with the increased thermal conductivity ratio of *k_s_^y^*/*k_s_^x^*, as shown in Figure 5a, but there is an opposite trend with increasing *k_s_^z^*/*k_s_^x^* in Figure 5b. The increasing *k_s_^z^* means the heat conduction increases along the *z*-direction more quickly, thus reducing the in-plane heat diffusion, which has a negative effect on the uniform temperature on the bottom surface of the substrate.

**Figure 6:**
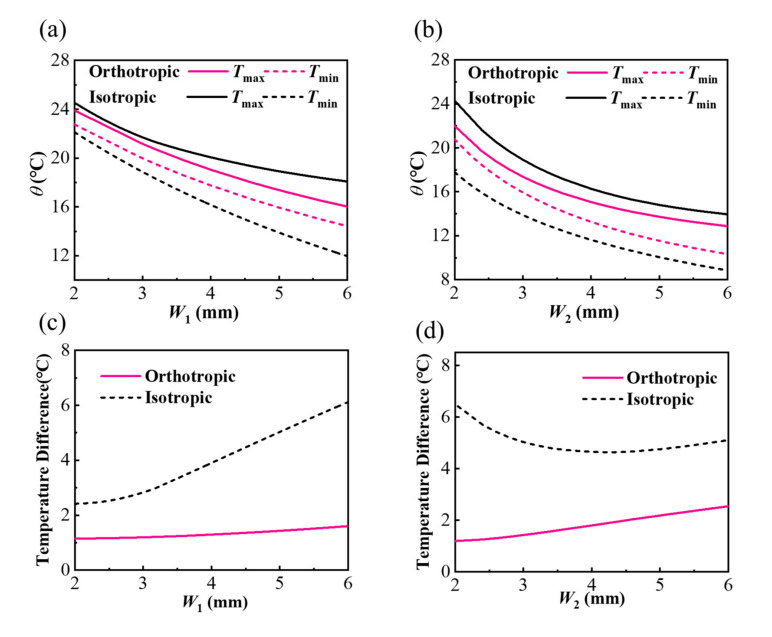
(**a**,**b**) The maximum (*T*_max_), the minimum (*T*_min_) temperature and (**c**,**d**) temperature difference on the bottom surface of the orthotropic and isotropic model with different vertical space *W*_1_ and horizontal space *W*_2_.

Figure 6 illustrates geometric effects of vertical space *W*_1_ and horizontal space *W*_2_ on the thermal properties of models with isotropic (*k_s_^x^* = *k_s_^y^* = *k_s_^z^* = 0.17 W/m/K) and orthotropic substrate (*k_s_^x^* = *k_s_^z^* = 0.17 W/m/K and *k_s_^y^* = 1.7 W/m/K). It can be observed from Figure 6a,b that the maximum (*T*_max_) and the minimum (*T*_min_) temperature decreases with the increasing spaces. Figure 6c shows that the temperature difference in the orthotropic model is lower than that in the isotropic model with different *W*_1_, which is the same as models with different *W*_2_ shown in Figure 6d. The result shows that the orthotropic substrate with larger *k_s_^y^* can help improve the temperature uniformity on the bottom surface with different spaces, *W*_1_ and *W*_2,_ of heat sources. When the *W*_1_ is increasing, the temperature uniformity of the orthotropic model is better than the isotropic model, as shown in Figure 6c. Meanwhile, there is an interesting trend in the isotropic model shown in Figure 6d that the temperature difference is not monotonic with the increasing *W*_2_, because the *T*_max_ drops faster than *T*_min_ in the isotropic model in the certain area of *W*_2_.

**Figure 7:**
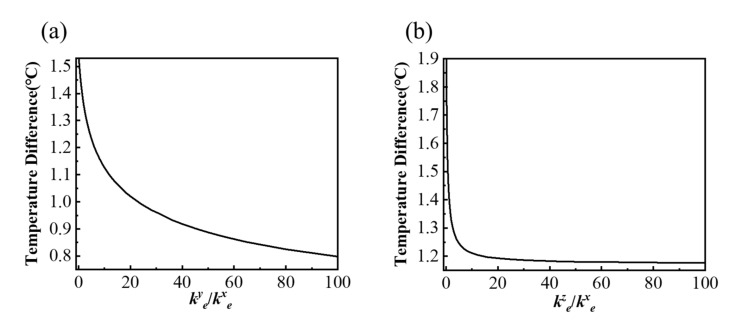
Effects of the orthotropic encapsulation with different ratios of (**a**) *k_e_^y^_/_k_e_^x^* and (**b**) *k_e_^z^_/_k_e_^x^* on the thermal uniformity of the bottom surface of the model.

The above research mainly focuses on the orthotropic substrate. In fact, the temperature distribution on the bottom surface of the substrate also depends on the thermal characteristics of the encapsulation. Therefore, the orthotropic encapsulation with different thermal conductivity ratios of *k_e_^y^*/*k_e_^x^* and *k_e_^z^*/*k_e_^x^* are investigated in Figure 7, where the substrate is selected as the isotropic substrate. It can be seen that the temperature difference decreases with the increasing *k_e_^y^*/*k_e_^x^* and *k_e_^z^*/*k_e_^x^*, because the improvement of the thermal conductivity of the encapsulation can effectively transfer heat to the top layer, thus reducing the heat flow in the substrate, which can help improve the uniform temperature of the bottom surface on the substrate. Additionally, the thermal conductivity *k_e_^z^* in encapsulation has a greater impact on the temperature uniformity on the bottom surface because the temperature difference falls faster than that with *k_e_^y^*, as shown in Figure 7.

## 4. Conclusions

In summary, this paper presents an analytical orthotropic heat conduction model with a serpentine heat source, which is validated by FEA. Unlike traditional heating components with a regular heat source shape and isotropic materials, the orthotropic flexible heater is used for uniform heating via adjusting the thermal conductivity in different directions. The temperature distribution on the bottom surface of the model is taken as the research object, which is the surface in contact with the target object. The results show that the higher thermal conductivity of substrate *k_s_^y^* along the vertical spacing of the heat source can effectively improve the temperature uniformity with different geometric spacing. On the contrary, improving the thermal conductivities of the substrate along other directions is ineffective. Additionally, the higher thermal conductivity of orthotropic encapsulation can obtain thermal uniformity as well. This study can be exploited in the design of a serpentine heater with the orthotropic model and provide easily interpretable guidelines to control the temperature distribution.
